# Stem Cells: Innovative Therapeutic Options for Neurodegenerative Diseases?

**DOI:** 10.3390/cells10081992

**Published:** 2021-08-05

**Authors:** Gabriele Bonaventura, Antonio Munafò, Carlo Maria Bellanca, Valentina La Cognata, Rosario Iemmolo, Giuseppe Antonino Attaguile, Rosaria Di Mauro, Giulia Di Benedetto, Giuseppina Cantarella, Maria Luisa Barcellona, Sebastiano Cavallaro, Renato Bernardini

**Affiliations:** 1 Institute for Biomedical Research and Innovation (IRIB), Italian National Research Council, 95126 Catania, Italy; gabriele.bonaventura@gmail.com (G.B.); valentinalacognata@hotmail.it (V.L.C.); iemmolo.rosario@gmail.com (R.I.); sebastiano.cavallaro@cnr.it (S.C.); 2 Department Biomedical and Biotechnological Sciences (BIOMETEC), Section of Pharmacology, University of Catania, 95123 Catania, Italy; uni315775@studium.unict.it (A.M.); uni318437@studium.unict.it (C.M.B.); peppettg@hotmail.it (G.A.A.); uni189440@studium.unict.it (R.D.M.); giulia.dibenedetto@studium.unict.it (G.D.B.); gcantare@unict.it (G.C.); 3 Department Pharmaceutical Science, Biochemistry Section, University of Catania, 95123 Catania, Italy; marsirin@gmail.com

**Keywords:** stem cell therapy, Alzheimer’s disease, Parkinson’s disease, amyotrophic lateral sclerosis, immunomodulation

## Abstract

Neurodegenerative diseases are characterized by the progressive loss of structure and/or function of both neurons and glial cells, leading to different degrees of pathology and loss of cognition. The hypothesis of circuit reconstruction in the damaged brain via direct cell replacement has been pursued extensively so far. In this context, stem cells represent a useful option since they provide tissue restoration through the substitution of damaged neuronal cells with exogenous stem cells and create a neuro-protective environment through the release of bioactive molecules for healthy neurons, as well. These peculiar properties of stem cells are opening to potential therapeutic strategies for the treatment of severe neurodegenerative disorders, for which the absence of effective treatment options leads to an increasingly socio-economic burden. Currently, the introduction of new technologies in the field of stem cells and the implementation of alternative cell tissues sources are pointing to exciting frontiers in this area of research. Here, we provide an update of the current knowledge about source and administration routes of stem cells, and review light and shadows of cells replacement therapy for the treatment of the three main neurodegenerative disorders (Amyotrophic lateral sclerosis, Parkinson’s, and Alzheimer’s disease).

## 1. Introduction

Stem cells, present in every living organism, are cells characterized by the unique ability to proliferate indefinitely by using a particular asymmetrical division, by self-renewal properties, and by the competence to drive their differentiation towards specific cell phenotypes in, virtually, all tissues [1]. In this perspective, the skill of stem cells to repair damaged tissues and restore physiological functions to diseased organs opens new scenarios for the treatment of pathological conditions for which current therapeutic options and respective clinical outcomes are quite poor or null [2]. In such a scenario, neurodegenerative disorders represent a promising target for stem cell-based therapy since the progressive loss of both neurons and glial cells unavoidably leads to irreversible damage in the central and peripheral nervous system. Multiple pathological mechanisms of damage, such as those occurring in Parkinson’s disease (PD), Alzheimer’s disease (AD), and Amyotrophic Lateral Sclerosis (ALS), have raised research interest for stem cell replacement therapy [3]. Some previous studies have reported that transplantation of stem cells into animal models of neurodegenerative diseases improved endogenous neuronal function by mediating remyelination, releasing trophic factors, and modulating inflammation [4,5,6,7,8]. To date, two different stem cell-based therapeutical approaches have been reported: the first one is based upon stimulation/boosting the endogenous neural progenitor cells to improve the release of trophic factors and growth molecules for tissue repair [9]; the second one points out grafting exogenous stem cells [10]. Here, we provide an update of the current knowledge about both source and administration routes of stem cells and review scientific literature regarding the use of stem cell replacement therapy for the treatment of the three main neurodegenerative diseases (i.e., AD, PD, and ALS).

## 2. Cellular Therapies from Different Cell Sources

**Figure 1:**
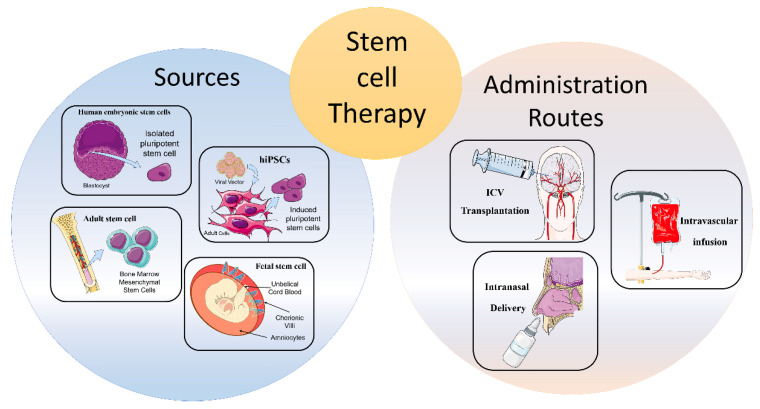
Schematic representation of stem cell therapy approach. Stem cell therapy is based on two macro-areas: sources of stem cell and routes of administration. Different tissue sources of stem cells have been examined for cell replacement therapies in neurodegenerative diseases. The most promising sources are showed in figure, all of them are characterized by specific advantages and limitation based on their origin and differentiation capacity. To date, only few routes of administration were tested and compared for efficacy. However, it is known that different administration routes can influence migration, distribution, and the amount of transplanted cells in the target area. In addition, administration routes influence dosage of stem cells and timing of cell delivery. Illustrations used elements from Servier Medical Art (https://smart.servier.com/, accessed on 15 July 2021).

Stem cells guarantee the physiological process of tissues regeneration and can be classified according to either their developmental competence (totipotent, pluripotent, or multipotent) or tissue origin (adult, fetal, and embryonal stem cells) [2]. In particular, totipotency refers to the ability to generate the entire spectrum of fetal cell types, including the placenta. Pluripotency refers to the ability to differentiate into the three embryonal germ layers (endoderm, mesoderm, and ectoderm), but not in the whole organism [11]. Finally, multipotency is the property to differentiate into specific cell phenotypes related to tissues of residence, even if it has been demonstrated that adult stem cells can cross boundaries and populate a different tissue [12]. The extremely limited and self-repairing capacity of adult neural tissue justifies the search for new sources of cells and the need for strategies of intervention in neurodegenerative diseases [12]. To this end, different tissue sources of stem cells have been examined in order to determine the most efficacious and productive method for cell replacement therapy in neurodegenerative disorders (Figure 1) [12].

### 2.1. Human Embryonic Stem Cells

Embryonic stem cells (ESCs) are isolated from the inner cell mass (ICM) of mammalian blastocysts. These cells show pluripotent properties and can easily differentiate into tissues from the three primordial germ layers (ectoderm, mesoderm, and endoderm), finally constituting the complete soma of the adult organism [13]. The principal therapeutic approach of human ESC (hESC) is based on the generation of specialized cells for the replacement of damaged tissue. Although this cell source is widely used in regenerative medicine to obtain human neuronal progenitors, several concerns associated with their use in clinical applications must still be addressed [2] including the risk of immune rejection, tumor formation, and genetic instability following a prolonged time in culture [14]. In addition, isolation of hESC lines from the ICM at the blastocyst stage obliges the destruction of the embryo, which has raised both ethical and political concerns.

### 2.2. Human-Induced Pluripotent Stem Cells

In 2007, Takahashi and Yamanaka introduced a new technology able to reprogram mouse somatic cells into pluripotent embryonic-like cells by transferring through viral vectors four transcription factors (Oct4, Sox2, Klf4, and c-Myc) responsible for pluripotency [15,16] and called them induced pluripotent stem cells (iPSCs). Given their pluripotent properties, it is possible to differentiate stable iPSC colonies in vitro toward neural progenitor cells via embryoid bodies [17], 3D dimensional cellular aggregates consisting of different cell types cultured in adherent monolayers, which, under precise culturing conditions, can produce homogeneous neuronal populations [18]. The introduction of iPSC technology has represented a breakthrough in stem cell research, since it avoids ethical issues and solves the problem of immune rejection in stem cell transplantation, bringing it closer to clinical application. However, the problem of low efficiency of reprogramming still needs to be overcome before its extensive application.

### 2.3. Fetal Stem Cell

Fetal stem cells can be isolated from a direct biopsy of the fetus or from fetal annexes (umbilical cord blood, term placenta, chorionic villi, and amniotic fluid) [19,20]. Among other tissue sources, these cells represent a relatively accessible and rich font of progenitors with therapeutic potential for regenerative medicine. Several studies have previously demonstrated some intermediate properties between embryonic and adult stem cells, such as the proliferative ability and the lack of immunogenicity [21]. An interesting source of human fetal mesenchymal stem cell (MSC) is the perivascular connective tissue of umbilical cords, the Wharton’s jelly [22]. These mesenchymal progenitors, defined as human umbilical cord perivascular cells (HUCPVCs), are able to exert significant proliferative effects on primary cultures of neurons and glial cells and have remarkable neuroprotective influence following transplantation into animal models of spinal cord injury and PD. Their paracrine potential was mainly expressed via the increase of human neutrophil-activating protein-2 (NAP-2), neurotrophin-3 (NT-3), basic fibroblast growth factor (bFGF), and glial-derived neurotrophic factor (GDNF) at the site of injury [23]. However, although working with umbilical cord blood appears to circumvent most of the ethical issues associated with research on fetal material, fetal stem cell research remains still in its infancy [24].

### 2.4. Adult Stem Cells

Adult stem cells, also known as somatic stem cells, are present in specialized niches of all tissues, where they act as key regulators of homeostasis, driving the cell fate between self-renewal and differentiation. Among this broad subgroup, both MSC and hematopoietic stem cells (HSC) have been widely investigated and applied for over 60 years in clinical practice paving the way for bone marrow (BM) transplantation [25]. Interestingly, it has been demonstrated that BM-MSCs are able to pass through the blood-brain barrier (BBB) after transplantation without altering the barrier’s structure and differentiate into neuron-like cells with neuroprotective properties due to the release of neurotrophic factors in the damage-site [26]. In this context, Yang et al. reported that the administration of BM-MSCs, overexpressing GDNF, displays neuroprotective effects in a rat model of intracerebral hemorrhage (ICH) [27].

## 3. Routes of Administration

Along with stem cell sources, administration routes can influence migration, distribution, and the amount of transplanted cells in the target area [28]. In addition, in accordance with different pathological conditions, the dosage of stem cells, as well as the timing of cell delivery, must be carefully considered [29]. To date, there are few studies directly comparing the efficacy of diverse transplantation routes; therefore, the optimum delivery route for specific cell types has not been determined [30].

### 3.1. Intracerebral or Intracerebroventricular Transplantation

Intracerebroventricular (ICV) of stem cells appeared for years to be the most precise delivery route for neural stem cells (NSC) implantation [30]. However, it showed different adverse reactions, such as motor exacerbations, syncope, seizures, and tumorigenicity. Therefore, the direct stereotaxic injection has been used less frequently in the first clinical trials [31,32].

### 3.2. Intravascular Infusion

Intravascular infusion, both intravenous and intra-arterial, represents a valid, safe, and less invasive alternative route of administration compared to ICV [33]. The peculiarity of this way of infusion is ability of exogenous cells to migrate towards the damaged tissue by passing the BBB [34]. Among the two routes of infusion, the intravenous administration seems less advantageous compared to the intra-arterial one, probably because of a trapping mechanism on the liver and lung area. Indeed, several studies reported a faster and more widespread cell distribution related to intra-arterial administration, since peripheral filter organs are overcome, thus leading to higher concentrations of exogenous stem cells in the target area [28,35,36,37]. However, these methods also present some disadvantages, including the standout thromboembolism microvascular occlusion, and injury exacerbation [38].

### 3.3. Intranasal Delivery

To date, the intranasal delivery route of administration represents a promising strategy to circumvent the BBB and to deliver drugs straight to the brain. This non-invasive way reduces the likelihood of adverse events compared to the intravascular one. Several studies have shown that stem/progenitor cells, administered intranasally, migrate to the brain through the nasal cavity and lead to positive findings in PD [39], malignant gliomas [40], and stroke [41,42,43]. In particular, transplanted cells migrate through the olfactory bulb, reach the cerebrospinal fluid (CSF) and, subsequently, the injured region. It has been demonstrated that chemokine gradients obtained from damaged cells can facilitate stem cell homing in target areas [44]. Conversely, disadvantages related to intranasal administration involve enzymatic degradation, low pH of the nasal epithelium, and individual variability that can lead to a lower release and efficacy in CNS [45]. Notwithstanding the limited studies obtained conducted so far, this route of administration may represent a potential avenue for the implantation of stem cells in the brain, specifically for neurological disorders.

## 4. Immunomodulation

Several studies reported evidence about the ability of stem cells to mediate tissue repair not only by a direct replacing of the damaged tissue but also acting as a key modulator of the local environment by influencing immune/inflammatory milieu and providing anti-apoptotic/cytoprotective effects on the resident cells [46,47]. This stem cell paracrine action is characterized by the release of a set of modulating molecules, including cytokines, chemokines, and growth/trophic factors, defined, as a whole, as secretome [48]. Secretome molecules can be released by stem cells through different mechanisms, including protein translocation, exocytosis, and extra cellular vesicles (EV), that act as carriers of bioactive molecules. The most widely studied EVs are exosomes, which are derived from endosomal membrane invagination, and micro-vesicles (MVs), which are generated from external budding of the plasma membrane regions full of ceramide and lipid rafts. In detail, MVs exhibit a diameter of 100–1000 nm with respect to 50–200 nm of exosomes [49]. A number of studies focusing on exosomes demonstrated their role in intercellular communications through the transfer of lipids, proteins, RNA, and miRNAs, thus regulating tissue physiological functions, immunomodulation, and tissue repair [50,51]. For example, Jarmalavičiūtė and colleagues [52] reported the anti-apoptotic effect of exosomes released by human dental pulp stem cells on dopaminergic neurons after treatment with 6-hydroxydopamine (6-OHDA), highlighting the protective effect on oxidative stress.

Stem cell-derived paracrine therapy could represent a new exciting pharmacological approach in avoiding immune compatibility and largely reducing the cost and time associated with expansion and maintenance of cell lines. Several studies have highlighted the contribution of stem cell transplantation in immunomodulation. For example, Wolbank et al. [22] reported specific modulatory properties of human placenta and amniotic membrane-derived mesenchymal stem cells (AMSCs) in regulating T-cell proliferation and dose-dependent inhibition of peripheral blood mononuclear cell-mediated immune responses. Kim et al. [53] reported a long-term beneficial effect of stem cells in a preclinical transgenic model of AD, suggesting a significant paracrine role in re-instructing the host compromised immune system and secreting amyloid beta-degrading enzymes. Kokaia et al. [54] reported the ability of stem cell to modulate T lymphocyte proliferation and dendritic cell maturation, evidencing a bilateral connection between engrafted stem cells and immune cells involved both in innate and adaptive immunity system. However, before achieving clinical applications, further investigations are required to identify the best stem cell source according to the paracrine potential, the large-scale production of specific paracrine molecules (proteins, lipids, small RNAs), the long-term safety, the bio-distribution, and the persistence of the therapeutic effects. In addition, EVs released by stem cells can be multi-functionalized to be used not only as therapeutic agents but also as vehicles for drug delivery [55].

## 5. Stem Cell Therapy for AD

**Figure 2:**
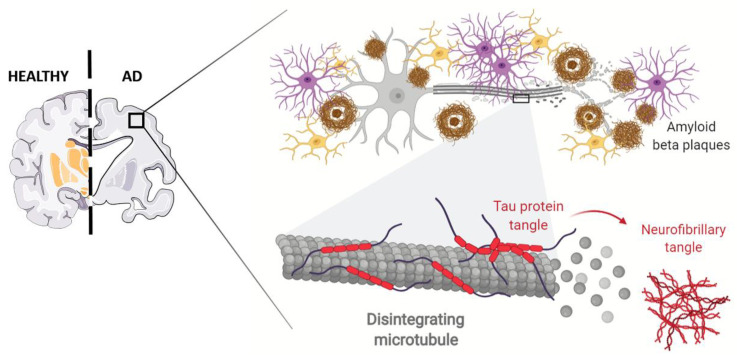
Schematic representation of Alzheimer Disease AD pathophysiology. This progressive neurodegenerative disorder is characterized by atrophy of brain regions, leading to a significant reduction in brain volume correlated to cognitive decline and memory deficits. The neuropathological hallmarks of AD are represented by extracellular senile plaques (the brown ring in the inlet), composed of amyloid-β (Aβ) peptide, followed by intracellular deposition of neurofibrillary tangles (NFTs) generated by hyperphosphorylated protein tau. In addition, glial (pink cells) and microglial (yellow cells) activation and subsequent inflammatory responses are thought to contribute to the neurodegenerative symptoms of AD. Adapted from “Alzheimer’s Brain (Disintegrating Microtubule)” by BioRender.com (2021). Retrieved from (https://app.biorender.com/biorender-templates/, accessed on 13 July 2021).

AD represents, arguably, the most significant social, economic, and medical crisis of our time [56]. This progressive neurodegenerative disorder, characterized by insidious onset and slow progression, leads to gradual dysfunction of cognition, memory, and learning in elderly people with huge implications for autonomy in daily life activities [57]. The pathogenesis of this progressive brain abnormality is multifactorial, being the result of interactions between age, a complex genetic profile, and intersecting environmental factors, including cardiovascular disease, traumatic brain injury, depression, and lower levels of education [57]. AD is first and foremost a condition of neuronal and synaptic loss throughout the brain, primarily affecting hippocampus and the basal forebrain networks, thereafter, progressing to brain cortex. Atrophy of these brain regions, leading to a significant reduction in brain volume, closely correlate to cognitive decline and memory deficits in these patients [58]. The acknowledged neuropathological hallmarks of AD are represented by extracellular senile plaques, composed of amyloid-β (Aβ) peptide, followed by intracellular deposition of neurofibrillary tangles (NFTs) generated by hyperphosphorylated protein tau [59]. Aβ is proteolytically derived from abnormal sequential cleavage of amyloid precursor protein (APP), carried out by β- and γ-secretase enzymes, resulting in extracellular accumulation and aggregation of protein fragments [60]. Tau is a microtubule-associated protein that plays a key role in axonal transport and neuronal structural support. The abnormal phosphorylation, decreasing tubulin binding capacity, causes both the microtubule disorganization and self-assembly into NFTs [61]. Although Aβ should be upstream of tau in AD pathogenesis and triggers its conversion from the normal to this toxic state, there is evidence that hyperphosphorylated tau enhances Aβ toxicity via a feedback loop [62]. In addition to these two specific proteins, microglial activation and subsequent inflammatory responses are thought to contribute to the neurodegenerative symptoms of AD [63]. Activated microglia produces several pro-inflammatory cytokines, including interleukin-1β (IL-1β) and tumor necrosis factor-α (TNF-α), that may contribute to neuronal dysfunction, injury, and loss [64]. Several theoretical hypotheses have been raised for elucidating the pathological mechanisms of AD, tau hypothesis, mitochondrial cascade hypothesis, oxidative stress hypothesis, and neuroinflammation hypothesis. Among these, amyloid-cascade hypothesis remained widely accepted as the centerpiece of AD pathology [65] (Figure 2).

Several drugs, focused on facilitating amyloid clearance or preventing amyloid production, have been developed and investigated in clinical trials with results far from satisfactory, having failed to improve cognitive and functional ability of AD patients [66]. On 7 June 2021, Food and Drug Administration (FDA) approved Aduhelm (aducanumab) [67], a human IgG1 anti-Aβ monoclonal antibody claiming to reduce, in a dose-and time dependent manner, β-amyloid plaques in AD patients, as assessed by Positron Emission Tomography (PET) studies [68]. Despite the reduction of Aβ-burden could provide a surrogate endpoint to predict a meaningful therapeutic benefit, clinical data failed to show a significant protection from cognitive and functional decline over considerable adverse events, leaving significant uncertainty about an acceptable risk/benefit profile of the drug [69]. Furthermore, a nine-year post-approval confirmatory study was committed in order to attempt a reassessment of the real efficacy of the treatment, also in view of its burdensome costs. The current available treatments include cholinesterase inhibitors donepezil, galantamine, rivastigmine, and N-methyl-D-aspartate (NMDA) receptor antagonist memantine. These agents preserve acetylcholine levels and prevent glutamate neurotoxicity, thus providing temporary symptomatic relief as palliative agents without affecting pathophysiological disease progression [70]. Due to the negative results of the drugs that had been expected to have clinical benefits and the progressive and devastating nature of AD, there would need to be a breakthrough therapy to satisfy the high unmet need of patients [71].

Recently, researchers are attempting a multifaceted approach that includes stem cells and neuro-regeneration to shed light on a better understanding of cellular mechanisms of neurodegeneration involved and to study the possibility of development of efficient cell-based therapies. Stem cell-derived neurons may provide a drug-screening platform to identify small molecules able to improve well-established cellular phenotypes linked to Aβ and tau pathologies [72]. In particular, iPSCs allow modeling sporadic AD, which accounts for the majority of cases, in order to stratify patients according to differential drug responsiveness [73]. The therapeutic potential of several cell types has been studied in AD animal models achieving promising results [74,75]. Indeed, the main purpose of stem-cell therapy in AD is to produce new neurons with the purpose of replace those lost or damaged during disease progression or, alternatively, to bring forth glial cells to protect neuronal cells from ongoing degeneration [76]. Furthermore, transplantation of stem cells in AD animal models can ameliorate cognitive impairment by reducing Aβ deposits through an enhanced alternative microglia activation as evidenced by increased levels of anti-inflammatory cytokines [77,78]. Likewise, stem cells can provide environmental support to residing existing neural networks secreting a variety of neurotrophic factors in affected areas, such as GDNF, nerve growth factor (NGF), and brain-derived neurotrophic factor (BDNF), to the extent that recent evidence suggests that beneficial effects of this therapeutic strategy depend instead in their paracrine signaling [79]. In addition, the efficacy of stem cell transplantation, both on promoting cognitive functions and on restoring learning defects, was confirmed by numerous research findings.

Despite promising results of preclinical data, numerous unresolved safety issues need to be overcome prior to transferring this technology from the bench to the bedside and human clinical trials are still in their infancy regarding stem cell therapy in AD (Table 1) [80]. The most-used cell type for this purpose is the MSCs (NCT01547689, NCT02672306 and NCT04228666), due to easy harvest, the possibility for intravenous transplantation, and lack of ethical issue, although key differences exist about cell number, dose number, and dose schedule [81].

In conclusion, although several challenges should be taken into account, stem cells carry enormous promise to enhance our understanding of AD molecular basis and provide a platform for discovery of novel therapeutic options. Since the clinical applications of stem cell technology are still in their early stages, this strategy is likely to become crucial and contribute significantly to the treatment of AD in the near future.

## 6. Stem Cell Therapy in ALS

**Figure 3:**
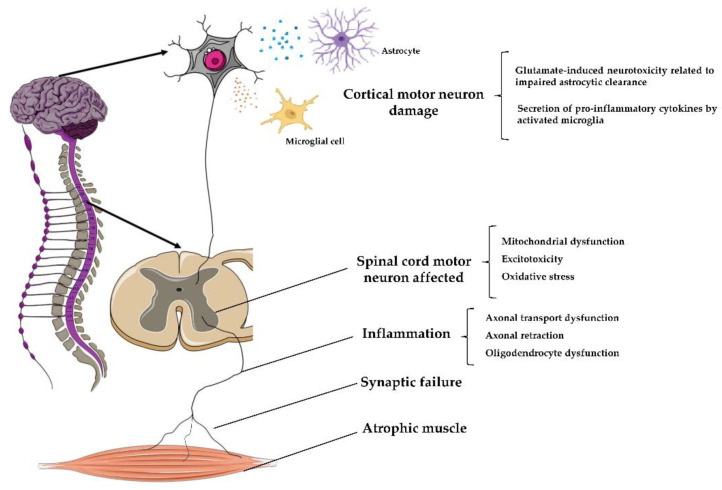
Schematic representation of Amyotrophic Lateral Sclerosis (ALS) pathophysiology. ALS is a progressive neurodegenerative disease, with a multifactorial etiology, characterized by motor neurons’ death in the motor cortex and in the spinal cord’s ventral horn. Furthermore, astrocytes are not able to support neuronal functions and impaired glutamate clearance leads to neuronal excitotoxicity, this, combined with the secretion of pro-inflammatory cytokines by predominant activated microglia, contributes to the development of motor neuron dysfunction. The ventral roots become thin with loss of large myelinated fibers in motor nerves leading to denervation atrophy in affected muscles. Illustrations used elements from Servier Medical Art (https://smart.servier.com/, 12 July 2021).

ALS, also known as Lou Gehrig’s disease, is a progressive neurodegenerative disease, with a multifactorial etiology, characterized by motor neurons’ loss in the spinal cord’s ventral horn and in the motor cortex [82]. The worldwide ALS prevalence and incidence rate are roughly 4.42/100.000 and 1.59/100.000, respectively, in males and females. In the early stages, this pathological condition is characterized by muscle weakness, contractions, stiffness, and loss of voluntary movement control, leading in the later stages, to a complete muscle paralysis (Figure 3). Death occurs approximately 3–5 years after the symptom onset, mainly because of respiratory failure [83]. In the last two decades, two different forms of ALS have been described: familial ALS (FALS) and sporadic ALS (SALS). Familial forms (5–10% of all cases) are characterized by an autosomal dominant inheritance associated with roughly 20 gene mutations, including superoxide dismutase-1 (*SOD1*), TAR DNA-binding protein (*TARDBP*), fused in sarcoma (*FUS*), amyotrophic lateral sclerosis 2 (*ALS2*), senataxin (*SETX*), janus-faced spatacsin (*SPG11*), vesicle-associated membrane protein-associated protein B (*VAPB*), and angiogenin gene (*ANG*) [84]. In addition, up to 50% of patients, correlated to familial forms of ALS, showed mutation of chromosome 9 open reading frame 72 (*C9orf72*), a gene also involved in cognitive dysfunction as frontotemporal dementia [85]. The SALS form (~90% of cases) is described as a complex multifactorial disorder, characterized by defects in protein aggregation, mitochondrial dysfunction, and oxidative stress, leading to excitotoxicity.

Today, no effective therapies were shown to provide a substantial clinical benefit for ALS patients. FDA approved only two treatments: riluzole, which prolongs median survival by about only 2 to 3 months [86], and edaravone, which slightly reduces the rate of decline in the early stages of disease [87,88]. In this context, stem cell therapy could represent a valid and alternative approach respect to the classical pharmacological therapy, by direct replacing dead/damaged cells, or by the releasing of factors that will provide neuroprotective effects, modulating pathogenetic pathways and modulating inflammation [89]. Different sources of stem cells have been investigated in preclinical and clinical trials for ALS to determinate the most efficacious and productive method, including ESCs, MSCs, immune system stem cells, NSCs/NPCs, and iPSCs. In addition to cell source selection, another prominent issue is the delivery method to obtain a minimally invasive injections strategy and an extensive cellular distribution along neuraxis [89]. For these reasons, several routes of administration have been examined in trials which have occurred over time. Several evidence reported that local brain transplantation or spinal cord injection may represent a useful via, particularly for NSCs/NPCs, since, by using other routes of administration, these cells could not penetrate into host CNS and, furthermore, undergo a degeneration process [90]. The advantage of stem cell local injections is based on releasing cells close to their target, allowing the diffusion of trophic and immunomodulatory factors and enhancing, in this way, the chance to obtain therapeutic effects. Following this purpose, a recent alternative way to administrate stem cells could be the use of stereotaxic devices MRI-guided [91] since it could be able to facilitate their specific local delivery.

Previous preclinical stem cell transplantation studies have been performed in mice and rats ALS model expressing mutant SOD1, reporting motor neurons degeneration, muscle atrophy and motor dysfunction. For example, Chen R. and Ende N. [92], by administering intravenously human umbilical cord blood (hUCB) into SOD1 mice, showed a delayed motoneurons degeneration and extended overall survival rate. Histological analysis of treated mice indicated that hUCBs have penetrated the brain and spinal cord parenchyma in regions with motor neurons degeneration expressing specific neural biomarkers, including glial fibrillary acidic protein (GFAP), nestin, and neuron-specific class III β-tubulin (TUJ1) [93]. Additionally, pro-inflammatory cytokines expression was lowered in the brain and spinal cord [94,95].

Regarding clinical trials, the first one approved by FDA employed NPCs in 18 affected ALS-patients with the scope to assess the safety of this kind of cells into the spinal cord [96]. Numerous clinical trials evaluated safety and feasibility of intraspinal, intrathecal and intracerebral MSCs transplantation; for instance, Mazzini et al. [97] experimented implantation of these cells into the dorsal spinal cord, showing no immediate or long-complications in a follow-up period of 9 years. Furthermore, other clinical trials are focused on the exogenous transplantation of NSCs due to valid data indicating the slowing down progression of ALS cells injection into subjects’ spinal cord [98]. Subsequently, we report the current clinical trials on stem cell therapy for ALS (Table 2). In conclusion, despite clinical applications are still in its infancy, the potential therapeutic effects of stem cell therapy in ALS represent a promising therapeutic strategy, acting through the modulation of mechanisms which can contribute to survival and functionality of resident cells.

## 7. Stem Cell Therapy in PD

**Figure 4:**
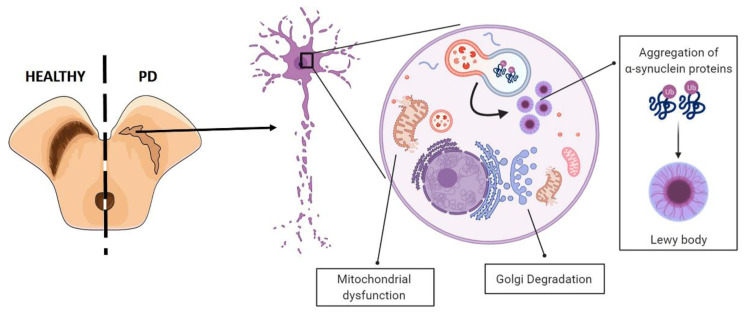
Schematic representation of Parkinson Disease (PD) pathophysiology. The main pathological hallmark of PD the progressive degeneration of striatal-projecting midbrain dopaminergic neurons of the ventral forebrain due to aggregation and accumulation of the protein α-synuclein (Lewy bodies), which cause mitochondrial disfunctions, endoplasmatic reticulum (ER) stress, and Golgi degradation. Illustrations used elements from Servier Medical Art (https://smart.servier.com/, 15 July 2021).

PD is one of the most frequent chronic neurodegenerative disorder affecting aged people [99,100]. This pathophysiological condition, either sporadic or familial, is characterized by the progressive degeneration of striatal-projecting midbrain dopaminergic neurons of the ventral forebrain, resulting in a reduced level of dopamine (DA) in the striatum area, leading to both motor symptoms, such as bradykinesia, rigidity, resting tremor, and postural instability, and cognitive alterations, including depression, dementia, hallucinosis, and sleep and sensory disorders [99,101]. The etiology of PD is still largely unknown, although it has been demonstrated that involves multifactorial factors, including genetics, environmental agents, and aging (Figure 4).

Despite the advances in the treatment of PD, there is still no specific cure for this condition. Conventional medications (e.g., levodopa) are highly effective in the early stage of PD in relieving motor symptoms, but systemic administration is often associated with adverse long-term side effects, such as drug-induced dyskinesia [102]. More recently, the transplantation of dopaminergic (DA)-cells into brains of PD patients for replacing degenerating neurons has gained new popularity due to successful generation of DA-producing cells from ESCs, NSCs, MSCs, iPSCs, or induced neurons converted directly form somatic cells (iN) [103]. The first lessons from cell replacement therapy for PD derive from a diverse array of open label trials and double-blind placebo-controlled trials conducted in order to analyze the efficacy of human fetal ventral mesencephalon (hfVM) grafting in the striatum of PD patients. Obtained results showed the proof-of-principle that fVM transplantation works and relieves tremor to some extent in most patients with long term clinical benefits, but, for a minority of subjects, the method failed to show a measurable benefit or, worse, it caused debilitating side effects and complications [104]. These highly variable outcomes have prompted a large-scale still ongoing multicenter open-label study called TRANSEURO (NCT01898390) projected to include a study cohort based upon careful analysis of previous trials, standardized and reproducible surgical placement protocols, and with long term follow-up using robust outcome measures (http://www.transeuro.org.uk/, accessed on 21 June 2021) [105]. Completion of TRANSEURO is expected at the end of 2021 [106].

A useful candidate stem-cell equivalent source might be represented by MSCs that have been shown to reduce dopamine depletion and rebuild the damaged striatal dopaminergic nerve terminal network in a PD animal model [98]. A previous pilot clinical trial performed MSC transplantations in the lateral ventricles’ walls of PD patients and reported promising functional recovery with no adverse effects and improved dyskinesias [107]. In addition, iPSCs are revealing a promising source and provide several advantages over human fVM. First, the primary cell source (blood cells or skin fibroblasts) for iPSC is easily and legally obtained worldwide; secondly, they can be immunologically compatible with the patient, given the ability to use patient’s own cells, or HLA-matched cells [108], and, thirdly, good manufacturing practice (GMP) standards for iPSC and DA precursor generation have already been developed yet [109]. Based on these efforts, the first clinical trial based on allogeneic transplantation, from HLA-matched, iPS-derived DA cells was launched in 2018 from the Japanese CiRA (Center for iPS Cell Research and Application). The main goals of this trial will be to evaluate the safety and efficacy of transplanting hiPSC-derived DA progenitors into the putamen of PD patients and, subsequently, to evaluate the safety and efficacy of using an immunosuppressive treatment (Tacrolimus) in engrafted patients [110]. Despite successes, ethical and legal issues make it difficult that the transplantation of stem cells to become a fully established therapy worldwide; therefore, researchers are looking for alternative sources of stem cells and optimized protocols with the aim to achieving mature functioning DA neurons [111]. Below, we present the active clinical trials that employ stem cell therapy for the treatment of PD (Table 3).

## 8. Conclusions

**Figure 5:**
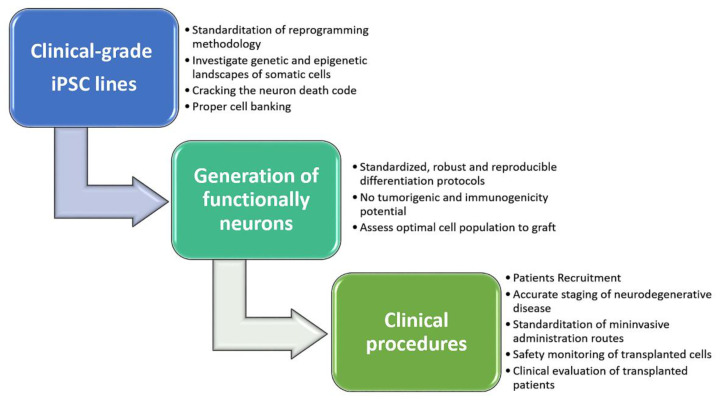
Schematic roadmap to guide successful clinical trials and maximize therapeutical potential of iPSC-based personalized cell replacement for patients with neurodegenerative diseases. Illustrations used elements from Servier Medical Art (https://smart.servier.com, 14 July 2021).

Stem cell technological improvements are opening new therapeutic perspectives for the treatment of several neurodegenerative diseases that, currently, lack of effective pharmacological therapy. The use of stem cells and their application in the replacement therapy is based both on their highly proliferative capability with peculiar differentiative properties, as well as on their cytoprotective effects, mediated by release of bio-molecules on the endogenous tissue environment. Following this standpoint, over the past few years, there has been progressive development of experimental procedures to generate human-derived neurons and glial cells, useful for cell replacement therapy. Although stem cell therapy could represent a ray of hope for patients suffering from neurodegenerative disorders, several concerns still need to be addressed to improve their application in the clinical setting (Figure 5). To this aim, special attention should be focused on the full control of proliferation and differentiation of the transplanted stem cells, with the purpose of generating completely functional new cells and promoting and maintaining their integration into endogenous neural networks. Regarding the paracrine modulatory effect of stem cells’ secretome, investigation of exosome content from different sources of stem cells, along with the possibility to genetically control contents and release of bio-molecules, could open new scenarios in the cell therapy approach.

In addition to these preeminent scientific issues, another substantial matter that needs to be evaluated is represented by the high running costs of stem cell therapy. Indeed, the production phases must comply with specific GMP standards [98], which involve appropriate materials, equipment, personnel, and facilities. Specifically, a study conducted in 2020, which estimated manufacturing and development costs for cell-based therapies, pointed out that the expenses for PSCs production are around 500,000 s [112]. Moreover, pre-clinical studies and clinical trials have considerable costs too; for instance, Geron Corporation, which developed the world’s first hESC product that entered clinical trials, had to invest about USD 200,000,000 in its hESC program, before FDA approval for a Phase I trial. Therefore, the development and production cost of this technologies will be reflected on the therapeutical option final price. Thereafter, is expected that the fees for stem cell-based therapy for neurodegenerative diseases will be between USD 30.000 and USD 100.000 for each patient [113]. These analyses reasonably prospect that, for now, these therapeutic approaches will be remain unavailable for a large number of patients but, with the progressive improvement of technological process, drug development costs will be reduced, allowing an increased number of subjects the access to these promising therapeutic options.

## Figures and Tables

**Table 1:** Current clinical trials on stem cell therapy for Alzheimer’s disease.

Title	Brief Summary	Intervention in Experimental Arm	Primary Outcome	Status	NCT Number
Phase 2b, Randomized, Double-Blind, Active-Controlled Study to Assess the Efficacy and Safety of AstroStem, Autologous Adipose Tissue Derived Mesenchymal Stem Cells, in Patients with Alzheimer’s Disease	This is a phase 2b randomized, double-blind, active-controlled study with 2 treatment arms, to compare the efficacy and safety of AstroStem versus donepezil treatment in patients with mild AD. Eligible patients diagnosed with AD within one year of the start of treatment will be enrolled. Patients who are randomized into the treatment group will be administered via intravenously AstroStem and donepezil placebo every 4 weeks from Week 0 to Week 16.	Autologous adipose tissue derived mesenchymal stem cells (AdMSCs) administered intravenously and donepezil placebo.	Change from baseline to Week 28 in the ADAS-Cog score.	Not Yet Recruiting	NCT04482413
Open-Label, Single-Center, Phase I/II Clinical Trial to Evaluate the Safety and the Efficacy of Exosomes Derived from Allogenic Adipose Mesenchymal Stem Cells in Patients with Mild to Moderate Dementia Due to Alzheimer’s Disease	The purpose of this study is to evaluate the safety and efficacy of Exosomes Derived from Allogenic Adipose Mesenchymal Stem Cells(MSCs-Exos)in subjects with mild to moderate dementia due to Alzheimer’s Disease.	Low dosage (5 μg) MSCs-Exos administrated for nasal drip twice a week for 12 weeks. Mild dosage (10 μg) MSCs-Exos administrated for nasal drip twice a week for 12 weeks. High dosage (20 μg) MSCs-Exos administrated for nasal drip twice a week for 12 weeks.	Number of participants with treatment-related abnormal laboratory values of liver or kidney function. Number of participants with treatment-related adverse events as assessed by Common Terminology Criteria for Adverse Events (CTCAE) v4.0.	Recruiting	NCT04388982
Phase I, Prospective, Open-label Trial to Evaluate the Safety, Tolerability and Exploratory Outcomes of Multiple Allogeneic Human Mesenchymal Stem Cells (HMSC) Infusions in Patients with Mild to Moderate Alzheimer’s Disease	The purpose of this interventional research study is to test the safety, possible side effects, and possible effectiveness of human mesenchymal stem cell (HMSC) infusions when given to people with a diagnosis of mild to moderate Alzheimer’s disease.	Participants in the hMSC treatment group will receive a total of 4 doses administered intravenously, once a week, every 13 weeks within a year period.	Incidence of any Treatment-Emergent Serious Adverse Events (TE-SAEs). All adverse events will be evaluated for relationship with the study intervention.	Recruiting	NCT04040348
Alzheimer’s Autism and Cognitive Impairment Stem Cell Treatment Study	The purpose of the study is to evaluate the use of autologous Bone Marrow Derived Stem Cells (BMSC) as a mean to improve cognitive impairment as occurs in Alzheimer’s Disease and other dementias and to improve behavior and socialization issues which occur in adult with autism spectrum disorder. The use of Near Infrared Light, in conjunction with the use of BMSC, will also be assessed.	Intravenous administration of BMSC Fraction (14cc). Intravenous administration of BMSC Fraction (14cc) combined with Near Infrared Light exposure. Intravenous administration of BMSC Fraction (14cc) combined with Intranasal BMSC Fraction.	Mini-Mental Status Exam (MMSE). The change from pretreatment baseline to each time point (1, 3, 6, and 12 months post-treatment) will be assessed. Autism Spectrum Quotient Exam. The change in scoring from pretreatment baseline to each time point (1, 3, 6, and 12 months post-treatment) will be assessed.	Recruiting	NCT03724136
Randomized, Double-blind, Placebo-controlled, Phase I / IIa Clinical Trial for Evaluation of Safety and Potential Therapeutic Effect After Transplantation of CB-AC-02 in Patients with Alzheimer’s Disease	The objective of the study is to evaluate the safety and the potential therapeutic effects of intravenous transplantation of placenta-derived mesenchymal stem cells (CB-AC-02) in patients with Alzheimer’ disease in two treatment groups.	Group1: CB-AC-02 administration on day 0. Group2: CB-AC-02 administration on day 0 and on week 4.	The safety and tolerability of treatment with CB-AC-02 will be assessed by analysis of adverse events, abnormal findings, and standard laboratory tests.	Recruiting	NCT02899091
Phase IIa Study of Allogeneic Human Mesenchymal Stem Cells in Subjects with Mild to Moderate Dementia Due to Alzheimer’s Disease	The purpose of the study is to assess the safety and tolerability of ischemia-tolerant allogeneic human mesenchymal stem cells (hMSCs) administered intravenously versus placebo to subjects with mild to moderate dementia due to Alzheimer’s disease. Secondarily, to assess the preliminary efficacy of hMSCs versus placebo in subjects with Alzheimer’s-related dementia.	Intravenous administration of allogeneic hMSCs and Lactated Riunger’s Solution.	Safety of allogeneic hMSCs administration by assessment of adverse events.	Recruiting	NCT02833792

**Table 2:** Current clinical trials on stem cell therapy for Amyotrophic Lateral Sclerosis.

Title	Brief Summary	Intervention in Experimental Arm	Primary Outcome	Status	NCT Number
Clinical Trial in Phase II of Intramuscular Infusion of Autologous Bone Marrow Stem Cells in Patients with Amyotrophic Lateral Sclerosis	The purpose of this study is to assess the positive effects of autologous bone marrow mononuclear cells (BMNC) injection on the natural loss of motor units and on the increase in the size of the motor unit that occurs in patients with ALS during the evolution of the disease.	Intramuscular infusion of autologous BMNC into the transverse abdominal (TA) muscle of one of the lower limbs versus intramuscular infusion of saline solution (placebo) in the TA muscle of the contralateral side.	Rate of serious and non-serious adverse events related to the use of bone marrow mononuclear cells in patients with Amyotrophic Lateral Sclerosis. D50 index obtained from stimulus intensity curves.	Not yet recruiting	NCT04849065
The Evaluation of the Effect of Wharton’s Jelly Mesenchymal Stem Cells (WJMSCs) on the Immune System of Patients with Amyotrophic Lateral Sclerosis	The objective of this study is to evaluate the safety of intrathecal administration of WJMSCs and the impact on the immune system of patients with Amyotrophic Lateral Sclerosis.	Three intrathecal administration of mesenchymal stem cells isolated from Wharton’s jelly.	Number of Serious Adverse Event of Special Interest (S)AESI, including meningitis, toxic encephalopathy encephalitis, high fever, and epileptic seizures not connected to conditions above.	Recruiting	NCT04651855
A Phase II Study of Intrathecal Autologous Adipose-derived Mesenchymal Stromal Cells for Amyotrophic Lateral Sclerosis	The purpose of this open label, Phase II multi-site clinical study is to determine the safety and efficacy of intrathecal treatment delivered to the cerebrospinal fluid (CSF) of mesenchymal stem cells in ALS patients every 3 months for a total of 4 injections over 12 months.	Autologous adipose-derived Mesenchymal Stromal Cells (aaMSCs) will be administered intrathecally at a single dose suspended in 5-10 mL Lactated Ringer’s. Reduced dose treatments will be allowed based on specific adverse events.	Number of adverse events recorded from the time of enrollment until the end of the follow-up period or, in the case of early withdrawal, to the time of study withdrawal.	Recruiting	NCT03268603
A Phase 1/2a Open-Label Study to Investigate the Safety of the Transplantation (by Injection) of Human Glial Restricted Progenitor Cells (hGRPs; Q-Cells^®^) Into Subjects with Amyotrophic Lateral Sclerosis (ALS): Assessment of Localized Therapeutic Activity by Blinded Observation and Lateral Transplantation (ALTA-BOLT)	This study is a non-randomized, open-label, partially blinded, sequential cohort, dose-escalation study designed to obtain preliminary data on the safety, tolerability, and early efficacy of hGRPs transplantation in subjects with ALS. Following an initial cohort receiving cell transplants unilaterally in the lumbar spinal cord, subsequent cohorts will receive escalating doses transplanted unilaterally in cervical spinal cord. Subjects and outcome measure assessors will be blinded to side of treatment.	Unilateral lumbar surgical transplantation of human cells of the glial lineage.	Safety measured by the number of therapy-related adverse events.	Not yet recruiting	NCT02478450
A Multicenter Phase I/II Clinical Trial, Randomized, Controlled with Placebo, Triple Blind to Evaluate Safety, and Indications of Efficiency of the Intravenous Administration of the Therapy With 3 Doses of MSC in Patients with ASL Moderated to Severe	This is a multicenter phase I/II randomized, controlled with placebo, triple blind clinical trial aimed to evaluate the safety of intravenous administration of 3 doses of autologous mesenchymal stem cells from adipose tissue in patients with Amyotrophic Lateral Sclerosis. Forty patients will be enrolled and randomized into one of four arms and the follow-up phase, from the cell infusion/placebo, will be 6 months.	Intravenous administration of MSCs at different doses.	Number of adverse serious unexpected reactions or not, attributable to the treatment. Complications in the place of the infusion. Appearance of new neurological effect not attributable to the natural progression of pathology.	Active, not recruiting	NCT02290886

**Table 3:** Current clinical trials on stem cell therapy for Parkinson’s disease.

Title	Brief Summary	Intervention in Experimental Arm	Primary Outcome	Status	NCT Number
Clinical Investigation of Transplantation of Neural Stem Cell-derived Neurons for the Treatment of Parkinson’s Disease	This is a prospective study to demonstrate the safety and efficacy of differentiated neurons-derived from adult CNS progenitor cells transplanted in selected patients with Parkinson’s disease.	Intracerebral stereotactic microinjections of cell suspension into basal ganglia structures	Evaluation of various aspects of Parkinson’s disease, including non-motor and motor experiences, by Unified Parkinson’s Disease Rating Scale (UPDRS) Motor scale	Not yet recruiting	NCT03309514
A Safety and Efficacy Study of the Effects of Mesenchymal Stem Cells (MSCs) Differentiated into Neural Stem Cells (NSCs) on the Motor and Non-motor Symptoms in People with Parkinson’s Disease	This study is predicted to confirm the short term and long-term safety outcomes of the treatment of PD patients with umbilical cord derived stem cells.	Allogenic Umbilical Cord derived stem cells injected intravenously to enrolled PD patients. Allogenic Umbilical Cord derived stem cells (MSCs) differentiated into neural stem cells (NSCs) injected intrathecally and intravenously to enrolled PD patients.	Safety and tolerability assessment by report of Treatment-Emergent Adverse Events (TEAEs) because of the injection	Recruiting	NCT03684122
Clinical Study of Stereotactic Transplantation of Human Amniotic Epithelial Stem Cells (hAESCs) in the Treatment of Parkinson’s Disease (PD)	The purpose of this study is to evaluate the safety and efficacy of stereotactic transplantation of hAESCs for Parkinson’s disease. These cells are derived from placental amnion donated after cesarean section in healthy women.	Stereotactic transplantation of hAESCs into lateral ventricles to Parkinson’s disease participants.	Safety and tolerability assessment by report of adverse events	Not yet recruiting	NCT04414813
Allogeneic Bone Marrow-derived Mesenchymal Stem Cells as a Disease-modifying Therapy for Idiopathic Parkinson’s Disease: Phase IIa Double-blind Randomized Placebo Controlled Trial	The purpose of this study is to select the safest and most effective number of repeat doses of allogeneic bone marrow-derived mesenchymal stem cell (MSC) infusions to slow the progression of Parkinson’s disease	Two treatment infusions of MSC cells and 1 placebo every 3 months Three treatment infusions of MSC cells and 1 placebo every 3 months	Safest number of effective doses of MSC as measured by the Part III of the Movement Disorder Society Unified Parkinson’s disease Rating Scale (MDS-UPDRS) scale at different times.	Recruiting	NCT04506073
A Randomized, Double-Blind, Single Center, Phase 2, Efficacy and Safety Study of Autologous HB-adMSCs vs Placebo for the Treatment of Patients with Parkinson’s Disease	This is a randomized, double-blind, single center, phase 2 study aimed to assess efficacy and safety of multiple Hope Biosciences adipose derived mesenchymal stem cells (HB-adMSCs) versus placebo for the treatment of Parkinson’s disease. The trial includes a screening period of up to 4 weeks, a 32-week treatment period, and a safety follow-up period of 20 weeks after the last investigational product administration.	HB-adMSCs will be administered intravenously to study participants	Evaluation of changes in MDS-Unified Parkinson’s Disease Rating Scale (MDS-UPDRS). Incidence of treatment-emergent Adverse Event (TEAEs). Incidence of special interest AE, including thromboembolic events, infections, and hypersensitivities. Laboratory values: CMP, CBC, and coagulation panel. Report of vital signs and physical examination.	Recruiting	NCT04928287

## Data Availability

No new data were created or analyzed in this study. Data sharing is not applicable to this article.
